# Differential phenotypic expression of a novel *PDHA1* mutation in a female monozygotic twin pair

**DOI:** 10.1007/s00439-019-02075-9

**Published:** 2019-10-31

**Authors:** Alejandro Horga, Catherine E. Woodward, Alberto Mills, Isabel Pareés, Iain P. Hargreaves, Ruth M. Brown, Enrico Bugiardini, Tony Brooks, Andreea Manole, Elena Remzova, Shamima Rahman, Mary M. Reilly, Henry Houlden, Mary G. Sweeney, Garry K. Brown, James M. Polke, Federico Gago, Matthew J. Parton, Robert D. S. Pitceathly, Michael G. Hanna

**Affiliations:** 1 Department of Neuromuscular Diseases, UCL Queen Square Institute of Neurology and The National Hospital for Neurology and Neurosurgery, London, UK; 2 Neuromuscular Diseases Unit, Department of Neurology, Hospital Clínico San Carlos, IdISSC, Madrid, Spain; 3 Neurogenetics Unit, The National Hospital for Neurology and Neurosurgery, London, UK; 4 Area of Pharmacology, Department of Biomedical Sciences, School of Medicine and Health Sciences, University of Alcalá, Alcalá de Henares, Spain; 5 Sobell Department of Motor Neuroscience and Movement Disorders, UCL Queen Square Institute of Neurology, London, UK; 6 Neurometabolic Unit, The National Hospital for Neurology and Neurosurgery, London, UK; 7 Oxford Medical Genetics Laboratories, The Churchill Hospital, Oxford University Hospitals NHS Foundation Trust, Oxford, UK; 8 UCL Genomics, UCL Great Ormond Street Institute of Child Health, University College London, London, UK; 9 Metabolic Unit, Great Ormond Street Hospital for Children NHS Foundation Trust and UCL Great Ormond Street Institute of Child Health, London, UK

## Abstract

Pyruvate dehydrogenase complex (PDC) deficiency caused by mutations in the X-linked *PDHA1* gene has a broad clinical presentation, and the pattern of X-chromosome inactivation has been proposed as a major factor contributing to its variable expressivity in heterozygous females. Here, we report the first set of monozygotic twin females with PDC deficiency, caused by a novel, de novo heterozygous missense mutation in exon 11 of *PDHA1* (NM_000284.3: c.1100A>T). Both twins presented in infancy with a similar clinical phenotype including developmental delay, episodes of hypotonia or encephalopathy, epilepsy, and slowly progressive motor impairment due to pyramidal, extrapyramidal, and cerebellar involvement. However, they exhibited clear differences in disease severity that correlated well with residual PDC activities (approximately 60% and 20% of mean control values, respectively) and levels of immunoreactive E1α subunit in cultured skin fibroblasts. To address whether the observed clinical and biochemical differences could be explained by the pattern of X-chromosome inactivation, we undertook an androgen receptor assay in peripheral blood. In the less severely affected twin, a significant bias in the relative activity of the two X chromosomes with a ratio of approximately 75:25 was detected, while the ratio was close to 50:50 in the other twin. Although it may be difficult to extrapolate these results to other tissues, our observation provides further support to the hypothesis that the pattern of X-chromosome inactivation may influence the phenotypic expression of the same mutation in heterozygous females and broadens the clinical and genetic spectrum of PDC deficiency.

**Electronic supplementary material:**

The online version of this article (10.1007/s00439-019-02075-9) contains supplementary material, which is available to authorized users.

## Introduction

The pyruvate dehydrogenase complex (PDC) is a large mitochondrial multienzyme complex that catalyses the oxidative decarboxylation of pyruvate to acetyl-CoA, a rate-limiting step for the aerobic oxidation of glucose in the brain and other tissues. PDC contains multiple copies of three catalytic components (E1 or pyruvate dehydrogenase, E2 or dihydrolipoamide acetyltransferase, and E3 or dihydrolipoamide dehydrogenase) and the non-catalytic E3 binding protein. E1 is a thiamine diphosphate-dependent enzyme formed by two α and two β subunits (abbreviated E1α and E1β), whereas E2 and E3 consist of a single type of polypeptide chain. PDC activity is modulated by phosphorylation and dephosphorylation of three serine residues of E1α performed by two enzymes, pyruvate dehydrogenase kinase (PDK) and phosphatase (PDP), which are also linked to the complex. All components of PDC are encoded by autosomal genes with the exception of E1α, encoded by the *PDHA1* gene in the X chromosome (De Meirleir et al. 2016; Patel et al. 2014).

PDC deficiency represents a common cause of primary lactic acidosis and neurological disease in infancy and early childhood, with more than 400 cases reported to date (Sperl et al. 2015). Although mutations affecting E1β, E2, E3, and E3 binding protein and the regulatory enzyme PDP have been described, most cases are caused by mutations affecting E1α (Patel et al. 2012; Sperl et al. 2015). The clinical spectrum of PDC-E1α deficiency is broad. In males, three main presentations are recognised: (a) neonatal lactic acidosis and encephalopathy, sometimes associated with brain malformations; (b) infantile or childhood-onset Leigh or Leigh-like syndrome; and (c) a childhood-onset milder/relapsing neurological disorder that often includes ataxia, dystonia, and peripheral neuropathy. Heterozygous females appear to have a distinct clinical presentation that frequently includes dysmorphic features and microcephaly, especially in neonatal forms, in addition to moderate or severe psychomotor delay, spastic di/quadriplegia, and epilepsy. Brain imaging may reveal cortical/subcortical atrophy, dilated ventricles, cysts, and corpus callosum agenesis. Lactic acidosis may be present (Barnerias et al. 2010; De Meirleir et al. 2016; DeBrosse et al. 2012; Imbard et al. 2011; Lissens et al. 2000; Quintana et al. 2010).

That males are hemizygous and all females reported thus far are heterozygous for *PDHA1* mutations partly explains the clinical differences between sexes (Brown et al. 1994; Dahl 1995; Sperl et al. 2015). However, phenotypic variability among females with the same or functionally equivalent mutations also exists, and the pattern of X-chromosome inactivation (XCI) has been proposed as an important factor contributing to this variability (Brown et al. 1994; Dahl 1995; Dahl et al. 1992; Matthews et al. 1993). Here, we report for the first time female monozygotic twins with PDC-E1α deficiency, caused by a novel missense mutation in exon 11 of *PDHA1*. Both twins presented with a similar, primarily neurological phenotype but showed clear differences in disease severity, residual PDC activities, and XCI ratios in blood. This observation broadens the clinical and genetic spectrum of PDC-E1α deficiency and provides support to the hypothesis that the pattern of XCI is a major determinant of the phenotype in heterozygous females.

## Methods

The female twins were clinically evaluated in the Myopathy clinic and the Highly Specialised Service for Mitochondrial Disease clinic of the National Hospital for Neurology and Neurosurgery, London, UK. The study was granted ethical approval by the University College London (UCL) and UCL Hospital Joint Research Office (REC 09/H0716/76). Laboratory, neurophysiological, and imaging studies and tissue biopsies were performed using standard diagnostic protocols. Spectrophotometric assays of mitochondrial respiratory chain enzyme complexes in homogenised snap-frozen muscle samples were performed as previously described (Hargreaves et al. 2002). Total genomic DNA extracts from peripheral blood leukocytes and skeletal muscle biopsy were obtained for diagnostic purposes and used for research following informed consent. Diagnostic testing for point mutations and large-scale rearrangements of the mitochondrial DNA (mtDNA) was performed by restriction enzyme analysis, long-range PCR and Southern blotting on DNA extracted from skeletal muscle (Hammans et al. 1991; Muqit et al. 2008). For additional methods on molecular genetic studies, see the online Supplementary material.

### Fibroblasts studies

Skin biopsies were performed following informed consent. Fibroblasts from the patients were established from skin explants. All cells were maintained at 37 °C and 5% CO_2_ under humidified conditions and cultured in high glucose Dulbecco’s Modified Eagle medium (Invitrogen) supplemented with 10% foetal bovine serum (Biowest), penicillin/streptomycin, and 0.05 mg/ml uridine. Overall PDC activity was measured after maximal activation with dichloroacetate, using [1-^14^C]-pyruvate as substrate as described previously (Wicking et al. 1986). In this assay, the activity of normal control fibroblasts is 0.6–0.9 of ^14^CO_2_ produced/mg protein/min. For immunocytochemical analysis, cultured fibroblasts were fixed and stained with antibodies to the PDC E1α subunit and E2 enzyme as described previously (Lib et al. 2002). The anti-E1α antibody was detected with a secondary antibody labelled with Alexa Fluor 594 (red). The anti-E2 antibody was labelled directly with Alexa Fluor 488 (green).

## Results

### Case reports

**Fig. 1:**
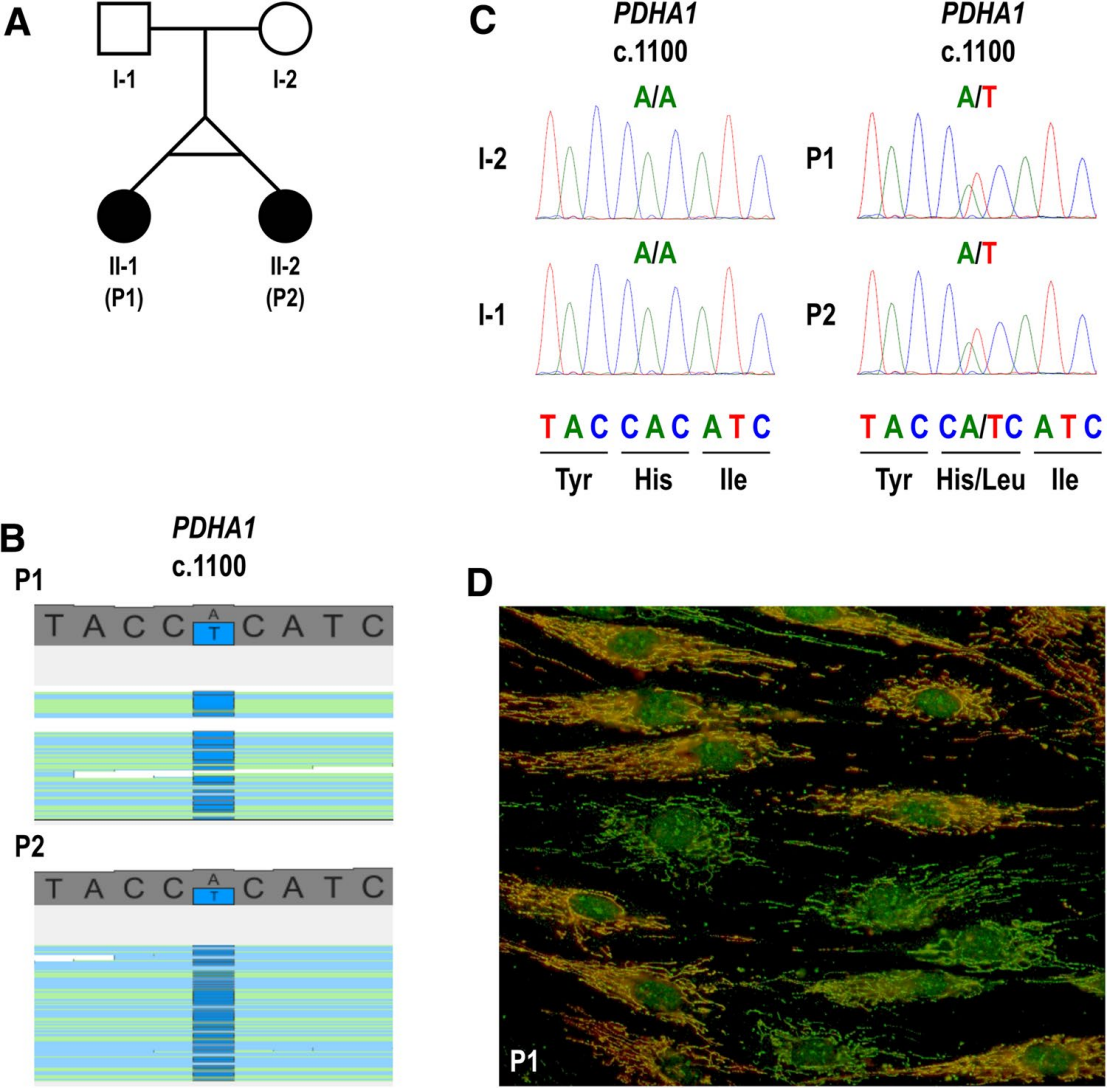
Family pedigree, DNA sequencing, and immunocytochemistry. **a** Family pedigree. **b** Visualisation of the heterozygous variant NM_000284.3 (*PDHA1*): c.1100A>T (p.His367Leu) on whole-exome sequencing. **c** Sanger sequencing electropherograms of nucleotide NM_000284.3 (*PDHA1*) c.1100. The heterozygous variant c.1100A>T is present in both twins and absent in their parents, confirming that the variant is de novo. **d** Mosaic expression of PDC E1α subunit in cultured fibroblasts from P1. Merged images of fibroblasts labelled with anti-E1α antibody (red) and anti-E2 antibody (green). Yellow cells express both the E2 component of the PDC (encoded in chromosome 11) and the E1α subunit, indicating that the active X chromosome in these cells contains the wild-type *PDHA1* allele. Green cells express the E2 component but are deficient in E1α subunit, indicating that the active X chromosome in these cells contains the mutant *PDHA1* allele

The female monozygotic twins were born to non-consanguineous parents by caesarean section for preeclampsia at 31 weeks of gestation (Fig. 1a). The second twin (P2) was briefly ventilated after delivery but there were no other major complications and both twins were discharged from the neonatal unit after 6 weeks. In subsequent months, they were noted to have mild global developmental delay and, at age 15 months, both became febrile and hypotonic 10 days after a vaccination, necessitating hospitalisation of P2. They lost developmental milestones after the episode, although subsequently gradually recovered. At age 3 years, P2 became confused and lethargic during a febrile illness and was admitted to the hospital again. This second episode prompted referral to a specialist clinic and, at age 5 years, both twins were diagnosed with Leigh syndrome on the basis of increased CSF lactate concentrations and abnormal neuroimaging (signal changes in the globi pallidi and peritrigonal white matter).

P2 continued to have episodes of acute encephalopathy triggered by infections, from which she recovered well. However, learning difficulties and impaired motor skills, coordination, and gait became apparent during the first decade of life, and repeated examinations revealed dysarthria, dysmetria, and dystonic posturing in the upper limbs, along with pyramidal tract signs in the lower limbs. She attended a special needs school and required a wheelchair for outdoor mobility during the teenage years. In adolescence, she suffered from three generalised tonic–clonic seizures (GTCS) during an encephalopathic episode and subsequently developed recurrent GTCS and seizures with impaired awareness and automatisms. Partial control of seizures was achieved with antiepileptic drug polytherapy. In her early 20s, following two further encephalopathic episodes with seizures, she developed behavioural changes and her overall motor function deteriorated. Coenzyme Q_10_ and quetiapine treatment was initiated at that time and symptoms slowly improved.

Aged 29 years, P2 was being treated with lamotrigine, levetiracetam, midazolam (as required), quetiapine, riboflavin, thiamine, l-carnitine, and coenzyme Q_10_. On examination, she was hypomimic and had moderate dysarthria and laterocollis to the left. Upgaze was markedly restricted, horizontal saccades were slow, and smooth pursuit was broken horizontally and vertically. Cranial nerve examination was otherwise normal. Tone was increased in limbs and there was mild weakness (MRC grade 4–4 +/5) distally in the upper limbs and proximally in the lower limbs. Deep tendon reflexes were all present (2/5) and plantar responses were extensor. She had a mild upper limb postural tremor and intention tremor with dysmetria, with an emphasis on the right side. There was also dystonic posturing and mild-to-moderate bradykinesia of the upper and lower limbs with left-sided predominance. Pinprick sensation, vibration sense, and joint position sense were normal. Romberg’s test was negative and she had a narrow-based, dystonic–spastic gait.

P1 had similar, but milder symptoms and clinical course compared to her sister. Her second and third encephalopathic episodes occurred at ages 14 and 19 years in the context of acute infections and she recovered completely. She developed seizures with impaired awareness and GTCS in her mid and late teenage years, respectively, but good seizure control was achieved with carbamazepine monotherapy. As with her sister, she had mild learning difficulties and, after the seizures started, began complaining of memory problems and perseverative behaviour. Motor function was retained in the upper limbs, but there were walking difficulties from early childhood. Examinations from the age of 10 revealed mild dysmetria and dystonia in the upper limbs and pyramidal tract signs in the lower limbs. She used a wheelchair outdoors since her early 20s.

Aged 29 years, P1 was being treated with carbamazepine, riboflavin, thiamine, l-carnitine, and coenzyme Q_10_. On examination, she had moderate dysarthria and mild laterocollis to the left. Upgaze was moderately restricted, saccades were mildly slowed, and smooth pursuit was broken horizontally and vertically. Cranial nerve examination was otherwise unremarkable. Tone was slightly increased in her lower limbs, but muscle strength was intact. Deep tendon reflexes were all present (3/5) and plantar responses were extensor. There was mild postural and intention tremor with mild dysmetria and dysdiadochokinesia in both upper limbs. There was also mild dystonic posturing of the upper limbs and mild bradykinesia of the left upper and lower limb. Pinprick sensation, vibration sense, and joint position sense were normal. Romberg’s test was negative. Gait was narrow based and mildly spastic.

### Diagnostic studies

Following the initial clinical diagnosis at age 5 years, the twins were reinvestigated 5 years later and again in their 20s to determine their precise biochemical and genetic aetiology. At age 10 years, CSF and plasma lactate concentrations were increased (Supplementary Table 1). Electroretinography and visual evoked potentials were normal. Brain MRI scans of both twins were described as showing bilateral signal changes in the globus pallidum, peritrigonal white matter, and cerebellum. A cyst of the velum interpositum was also observed in P1. Needle muscle biopsy revealed type 2 fibre atrophy in P1 and slightly increased amounts of fine lipid droplets within the muscle fibres in the two twins. Histochemical staining for mitochondrial enzymes was normal. Skin fibroblasts were obtained for biochemical analysis, but failed to grow in culture.

Between the ages of 17 and 28 years, routine blood tests were repeatedly normal except for mildly raised creatine kinase (190–210 IU/L; upper limit of normal 140 IU/L) and plasma lactate (Supplementary Table 1). Plasma ammonia, amino acids, and acyl-carnitines and urine organic acids were all within normal limits. White cell ubiquinone levels and lysosomal enzymes activities were normal. ECG and echocardiography showed no abnormalities. Brain MRI scans revealed bilateral lesions in the basal ganglia, peritrigonal white matter, and cerebellum that were more evident in P2 than P1 (Supplementary Fig. 1). Interictal EEG recordings revealed no signs of epileptiform activity. Brainstem evoked responses were normal in P1 but poorly formed in P2, suggesting either VIII cranial nerve or brainstem involvement. Further neuro-otological evaluation of both twins revealed impairment of ocular smooth pursuit, optokinetic responses, and vestibulo-ocular reflex suppression, consistent with cerebellar dysfunction. In P2, nerve conduction studies showed mildly decreased conduction velocities in the upper and lower limbs and mildly reduced sensory amplitudes in the lower limbs. Similar but milder changes with a patchy distribution were observed in P1 (Supplementary Tables 2 and 3). Quadriceps muscle biopsy of P1 at age 23 years only revealed features of denervation with reinnervation. The activity of mitochondrial respiratory chain complexes I, II + III, and IV was normal.

### Genetic and biochemical studies

Based on the phenotype and MRI features suggestive of Leigh syndrome, P1 was screened for common point mutations (m.3243A>G, m.8344A>G, and m.8993T>G/C) and large-scale rearrangements of the mtDNA extracted from muscle, which resulted negative. Whole-exome sequencing (WES) was subsequently performed in both patients. Their exomes were 93% concordant, supporting monozygosity of the twin pair. Analysis focused on nonsynonymous, splice-site and coding indel variants with a minor allele frequency < 0.1% in the Exome Aggregation Consortium dataset (ExAC; http://exac.broadinstitute.org/). From a total of 359 (P1) and 480 (P2) variants that met these filtering criteria, nine of them were in genes listed in MitoCarta, an inventory of genes encoding proteins with probable mitochondrial localisation (Supplementary Table 4) (Calvo et al. 2016). The nine candidate variants were individually reviewed and classified on the basis of gene-associated phenotypes and mode of inheritance (Supplementary Table 5). Only the heterozygous missense change NM_000284.3 (*PDHA1*): c.1100A>T (p.His367Leu) affected a gene known to cause lactic acidosis and/or Leigh syndrome, and was therefore considered the most plausible disease-causing variant (Fig. 1b). The presence of this heterozygous variant was confirmed by Sanger sequencing in both twins, but it was not detected in their parents, indicating that it had arisen de novo (Fig. 1c).

**Fig. 2:**
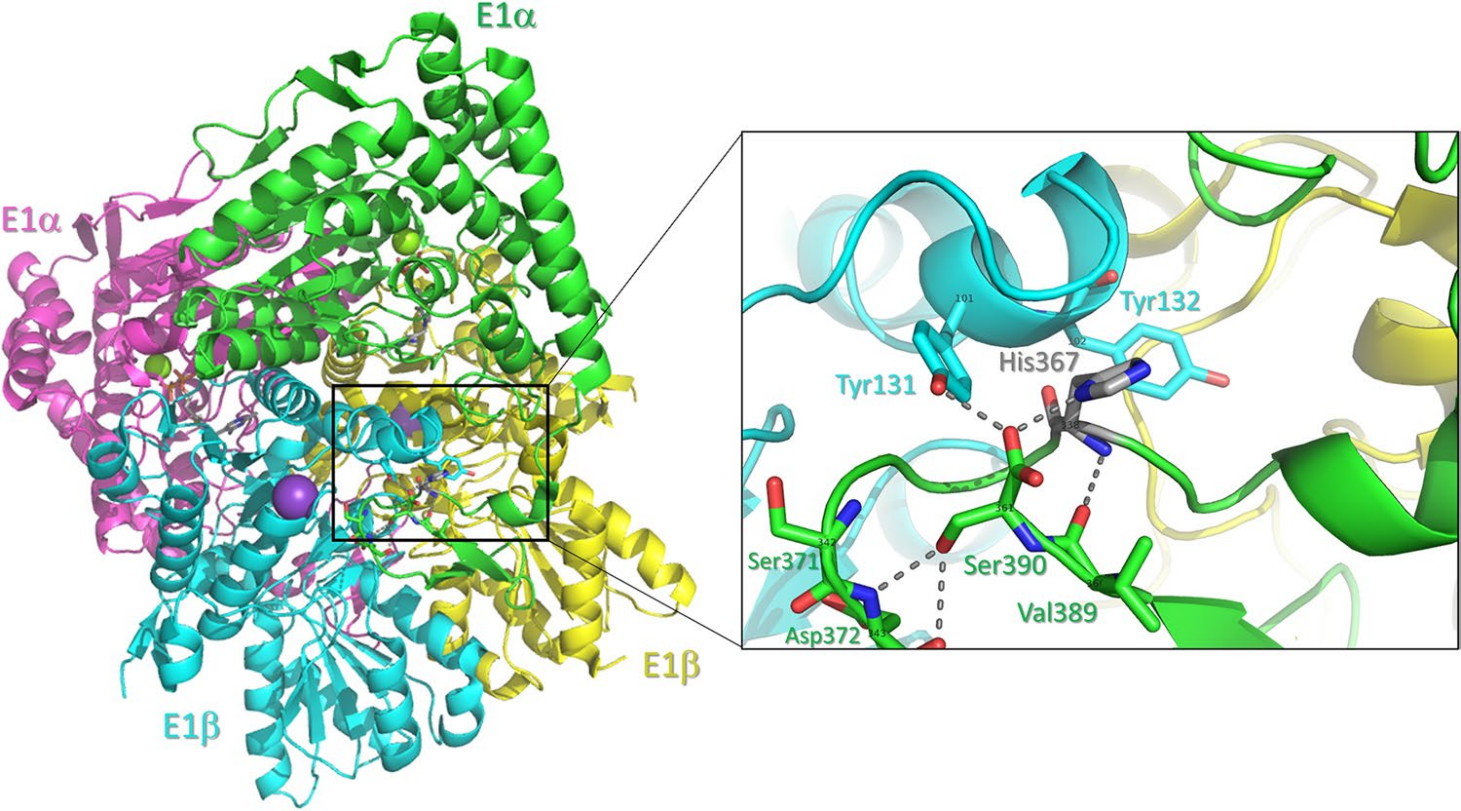
Three-dimensional structure of human pyruvate dehydrogenase (E1 component) of PDC. The four subunits are arranged tetrahedrally as a dimer of dimers and the tetramer possesses a 2-fold symmetry axis. The inset shows the location of p.His367 (grey sticks) at a loop position of E1α that is found at the interface with E1β. Hydrogen bonds are displayed as dashed lines. The small-sized labels on top of C-alpha carbons denote the PDB numbering scheme (as per entry 1NI4)

The c.1100A>T variant, located in exon 11 of *PDHA1*, is not reported in the Genome Aggregation Database (gnomAD; https://gnomad.broadinstitute.org/), but affects the same nucleotide and amino acid as c.1100A>C (p.His367Pro), which is registered in ClinVar as likely pathogenic based on two independent submissions (ClinVar accession number RCV000493915.1). c.1100A>T (p.His367Leu) is predicted as being deleterious by most of the pathogenicity prediction tools used (Supplementary Table 6) and affects an evolutionarily conserved histidine residue (Supplementary Fig. 2). Analysis of the structural changes caused by the p.His367Leu substitution shows a potential alteration of the conformation of the C-terminus of E1α and its interaction with E1β within the E1α_2_β_2_ heterotetramer. This is so because the side chain of p.His367 is involved in a direct hydrogen bond with the C-terminal carboxylate of p.Ser390 in the same E1α subunit; this carboxylate, in turn, hydrogen bonds to the side chain of p.Tyr131 in the E1β subunit (Fig. 2). The first hydrogen bond cannot exist in the p.His367Leu variant and the second one is likely to be weakened in the face of competing interactions with water. In addition, the hydrophobic side chain of p.Leu367 would tend to associate with the side chain of p.Leu137 in the E1β subunit (see Supplementary Figs. 3, 4 and 5 for details).

To confirm the functional impact of the variant, we measured the overall activity of PDC in cultured fibroblasts, which was moderately reduced in P1 and markedly reduced in P2 (Table 1). Immunocytochemical staining of E1α in fibroblasts revealed a mosaic pattern that appeared to correlate well with the residual activity of the enzyme in each case (Fig. 1d and Table 1). To address whether differences in disease severity between the two twins could be explained by differences in the pattern of XCI, we determined this pattern in DNA extracted from peripheral blood by assessing the methylation status of two CCGG sites located < 100 bp upstream a polymorphic CAG repeat within the androgen receptor (AR) gene. After restriction digestion using the methylation-sensitive HpaII enzyme, the CAG repeats on the inactive (methylated, undigested) X chromosomes were PCR amplified. Peak heights (signal intensity) of the resulting PCR products were determined by fragment analysis and the relative proportion of the two inactive X chromosome CAG alleles was calculated (see supplementary methods for details). Results were consistent with a skewed pattern of XCI in P1 (> 75–80% inactivation of the same allele) (Minks et al. 2008), but not in P2 (Table 1 and Supplementary Table 7). Analysis of CAG repeat sizes in the mother indicated that the maternal allele was preferentially active in both twins.

**Table 1:** Pyruvate dehydrogenase complex activity in cultured fibroblasts and X chromosome inactivation pattern in DNA extracted from peripheral blood

Patient	PDC activity^a^ (nmol/mg protein/min)	Immunoreactive E1α	XCI pattern^b^
P1	0.46	Moderate reduction	76:24
P2	0.17	Marked reduction	55:45

*E1α* E1α subunit of the E1 component of the pyruvate dehydrogenase complex, *PDC* pyruvate dehydrogenase complex, *XCI* X chromosome inactivation

^a^Range in normal controls: 0.6–0.9 nmol/mg protein/min

^b^Ratio of the two PCR-amplified CAG repeat alleles within the inactive X chromosomes

## Discussion

This report describes the first set of female monozygotic twins with PDC-E1α deficiency. Monozygosity was established on the basis of physical similarities and concordance of WES data. Both twins presented with a primarily neurological disorder that included mild developmental delay and learning difficulties, episodes of hypotonia or encephalopathy triggered by vaccination and infections from early childhood, and later onset of epilepsy and signs of pyramidal, extrapyramidal, and cerebellar dysfunction leading to slowly progressive motor impairment. Brain MRI revealed lesions similar to those seen in Leigh syndrome that were more symmetrically distributed in P2 than in P1. They had no dysmorphic features and no history of lactic acidosis, although their plasma lactate levels were often mildly increased and associated with a lactate:pyruvate ratio < 20. This clinical picture was consistent with previous descriptions of heterozygous females with PDC-E1α deficiency, but the impossibility to perform enzymatic assays at age 10 years due to failure of fibroblast culture caused a significant delay in the diagnosis. The molecular diagnosis was ultimately achieved using WES and the pathogenicity of the newly identified de novo mutation supported by biochemical and immunocytochemical studies.

PDC-E1α deficiency has considerable allelic heterogeneity with over 100 mutations, mostly missense mutations and small in-frame and frameshift indels, identified throughout the entire coding sequence of *PDHA1* except exon 2 (Imbard et al. 2011; Lissens et al. 2000; Patel et al. 2012; Quintana et al. 2010). Missense mutations are more common than indels and tend to localise to exons 3–9, whereas most indels are localised to exons 10–11. Hemizygous males, in whom the defective enzyme is expressed in all cells and the residual PDC activity depends on the effect of the mutation, frequently have missense mutations that allow the production of a partially functioning enzyme. In contrast, heterozygous females, in whom the defective enzyme is only expressed in a proportion of cells, can tolerate more severe mutations and have frameshift mutations more often than males. Heterozygous females harbouring missense mutations may be asymptomatic or have milder clinical phenotypes and survive into the second or third decades of life, as illustrated by the present and other cases (Cameron et al. 2004; Fujii et al. 1994; Lissens et al. 1999; van Dongen et al. 2015; Willemsen et al. 2006). However, they can also be severely affected (Cameron et al. 2004; Patel et al. 2012; Quintana et al. 2010; Willemsen et al. 2006), which likely reflects the differential effect of each mutation on E1α structure and function (Imbard et al. 2011; Korotchkina et al. 2004). In addition, heterozygous females can exhibit variable expression of the same missense mutation probably due to interindividual differences in the pattern of XCI (Brown et al. 1994; Dahl 1995; Dahl et al. 1992; De Meirleir et al. 1993; Fujii et al. 1996; Otero et al. 1998).

The novel missense mutation we detected in exon 11 of *PDHA1* (p.His367Leu) resides close to the C-terminal region of E1α, where other point mutations (e.g. p.Arg378Cys, p.Arg378His, and p.Trp383*) have been previously identified in female patients (Barnerias et al. 2010; Chun et al. 1995; Fujii et al. 1996; Willemsen et al. 2006). Structural analysis of p.His367Leu indicates a potential deleterious effect of this amino acid substitution on the conformation of the C-terminus of E1α and its interaction with E1β. These changes result in E1α_2_β_2_ heterotetramer instability in neighbouring regions that may lead to increased degradation of the unassembled subunits. This possibility is consistent with the observation of reduced steady-state levels of immunoreactive E1α in a proportion of cultured fibroblasts from both twins. A similar pathogenic mechanism has been proposed for the recurrent mutation p.Arg378His (Korotchkina et al. 2004), which severely impairs E1 function in vitro and is associated with reduced or undetectable amounts of E1α and E1β in patient-derived fibroblasts despite normal levels of E1α mRNA (Hansen et al. 1991; Tripatara et al. 1999; Wexler et al. 1997). E1α deletion mutants lacking one to four of the C-terminal amino acids (including p.Ser390, which forms a hydrogen bond with p.His367) (Korotchkina et al. 2004) also show decreased amounts of E1α and E1β despite normal transcription and mitochondrial import of E1α, supporting that the integrity of the C-terminus of E1α is essential for the stability or assembly of the E1 enzyme (Seyda et al. 2000).

Although monozygotic, the twins exhibited different disease severity with P1 being less severely affected than P2. This clinical observation correlated well with residual PDC activities in cultured skin fibroblasts, approximately 60% of mean control values in P1 and 20% in P2, and the corresponding reduction of immunoreactive E1α. Analysis of WES data did not reveal mutations involving other components of the PDC in either twin. Therefore, we considered the possibility of constitutional unbalanced XCI favouring the wild-type allele in P1 and/or the mutant allele in P2 as the mechanism underlying their phenotypic differences. Using the AR assay in DNA extracted from peripheral blood, we detected a XCI ratio close to 50:50 in P2, indicating similar degree of activation of each X chromosome. In P1, in contrast, there was a significant bias in the relative activity of the two X chromosomes with a ratio of approximately 75:25. Based on these results and the fact that the proportion of normal and E1α-deficient fibroblasts appear to remain constant in culture (Brown et al. 1990), it is possible that XCI skewing was also present in fibroblasts from P1, favouring the expression of wild-type E1α and, consequently, of fully functional PDC. It is well known that skewed XCI with ratios ≥ 80:20 biased towards the expression of the wild-type allele can lead to normal PDC activities in fibroblast from female patients (Brown and Brown 1993; Matthews et al. 1994; Willemsen et al. 2006). However, XCI ratios of 70:30–80:20 have also been associated with PDC activities of 60–65% of mean control values, both determined in fibroblasts (Brown and Brown 1993), similar to what we observed in P1 in different tissues.

We believe that differences in the pattern of XCI in the twins is the most plausible explanation for the observed phenotypic differences, and the results of the AR assay in blood provide indirect support for this hypothesis. A good level of concordance in the XCI pattern between haematopoietic tissues and brain has been demonstrated in some female individuals (Bittel et al. 2008). If this were the case in P1, skewed XCI biased towards the wild-type allele in brain might account for her milder neurological disease. Nevertheless, due to inter- and intra-tissue variations in the pattern of XCI, we cannot exclude other scenarios such as skewed XCI favouring the mutant allele in fibroblasts or brain in P2, or variations in the XCI mosaic across different neuroanatomical regions in P1. The latter possibility has been observed in post-mortem brain tissue from one individual with Rett syndrome (Gibson et al. 2005), an X-linked neurodevelopmental disorder that mostly affects females, as well as in genetically engineered female mice expressing fluorescent reporters that allow visualisation of XCI at cellular resolution (Wu et al. 2014). Of note, lesion distribution on brain MRI was patchier in P1 than in P2, which might reflect topographic fluctuations in the pattern of XCI in P1, although we cannot discount the influence of other epigenetic or environmental factors on disease expression.

Distinct expression of X-linked disorders in monozygotic twin females has been widely reported in the literature (Bennett et al. 2008; Costa et al. 1997; De Gregorio et al. 2005; Johnston-MacAnanny et al. 2011; Tiberio 1994; Watkiss et al. 1994; Willemsen et al. 2000). However, with a few exceptions (Devys et al. 1992; Ishii et al. 2001; Mittal et al. 2011), most reported twin pairs exhibited discordant phenotypes, i.e. only one twin was clinically affected and the other twin was asymptomatic, with or without paraclinical evidence of heterozygous carrier status (e.g. reduced α-galactosidase activity in Fabry disease or factor VIII procoagulant activity in haemophilia A) (Bennett et al. 2008; Redonnet-Vernhet et al. 1996). In contrast, both members of the twin pair described here were clinically affected. PDC plays a pivotal role in energy production from glucose in all metabolically active tissues, especially in the brain, where it operates at high levels of activity even during fasting states (Siess et al. 1971). Consequently, even relatively mild reductions in PDC activity in heterozygous females or mosaic individuals may have deleterious effects on normal brain development and function (Coughlin et al. 2010; Ridout et al. 2008). This most likely explains why a significant number of heterozygous females with PDC-E1α deficiency manifest a severe neurological phenotype and the co-occurrence of the disease in both twins.

## Electronic supplementary material

Below is the link to the electronic supplementary material.

Supplementary Material (DOCX 74 kb)

Supplementary Figures Legends (DOCX 25 kb)

Supplementary Figure 1 (PNG 13078 kb)

Supplementary Figure 2 (PNG 347 kb)

Supplementary Figure 3 (PNG 1984 kb)

Supplementary Figure 4 (PNG 9571 kb)

Supplementary Figure 5 (PNG 1256 kb)

〹

## Data Availability

Data sharing not applicable to this article as no datasets were generated or analysed during the current study.

## Abbreviations

E1α: α Subunit of the E1 component of the pyruvate dehydrogenase complex
E1β: β Subunit of the E1 component of the pyruvate dehydrogenase complex
GTCS: Generalised tonic–clonic seizures
PDC: Pyruvate dehydrogenase complex
ULN: Upper limit of normal
WES: Whole-exome sequencing
XCI: X-chromosome inactivation
