# Supplementary material for: Differential phenotypic expression of a novel PDHA1 mutation in a female monozygotic twin pair
Source: Hum Genet. 2019 Oct 31;138(11):1313–22. doi: 10.1007/s00439-019-02075-9 (PMC6874639; doi:10.1007/s00439-019-02075-9)
Supplement: Supplementary file 1 — Supplementary Material (DOCX 74 kb) [file 439_2019_2075_MOESM1_ESM.docx]

**Supplemental data to:**

**Differential phenotypic expression of a novel *PDHA1* mutation in a female monozygotic twin pair**

Alejandro Horga^1,2^, Catherine E Woodward^3^, Alberto Mills^4^, Isabel Pareés^5^, Iain P Hargreaves^6^, Ruth M Brown^7^, Enrico Bugiardini^1^, Tony Brooks^8^, Andreea Manole^9^, Elena Remzova^6^, Shamima Rahman^10^, Mary M Reilly^1^, Henry Houlden^1,9^, Mary G Sweeney^3‡^, Garry K Brown^7^, James M Polke^3^, Federico Gago^4^, Matthew J Parton^1^, Robert DS Pitceathly^1*^, Michael G Hanna^1*^.

(**1**) MRC Centre for Neuromuscular Diseases, UCL Queen Square Institute of Neurology and the National Hospital for Neurology and Neurosurgery, London, UK. (**2**) Neuromuscular Diseases Unit, Department of neurology, Hospital Clínico San Carlos, Madrid, Spain. (**3**) Department of Neurogenetics, the National Hospital for Neurology and Neurosurgery, London, UK. (**4**) Area of Pharmacology, Department of Biomedical Sciences, School of Medicine and Health Sciences, University of Alcalá, Alcalá de Henares, Spain. (**5**) Sobell Department of Motor Neuroscience and Movement Disorders, UCL Institute of Neurology, Queen Square, London, UK. (**6**) Neurometabolic Unit, the National Hospital for Neurology and Neurosurgery, London, UK. (**7**) Oxford Medical Genetics Laboratories, Oxford University Hospitals NHS Foundation Trust, The Churchill Hospital, Oxford, UK. (7) UCL Genomics, University College London, Institute of Child Health. (**8**) Department of Molecular Neuroscience, UCL Institute of Neurology, Queen Square, London, UK. (**9**) UCL Genomics, University College London, Institute of Child Health and UCL Great Ormond Street Institute of Child Health, London, UK. (**10**) Metabolic Unit, Great Ormond Street Hospital for Children, NHS Foundation Trust, London, UK. (**‡**) Deceased 26^th^ September 2017. *These authors contributed equally to the study.

**This file includes:**

Supplemental Tables 1-7

Supplemental methods

| **Supplementary Table 1**. Lactate and pyruvate concentrations in plasma and cerebrospinal fluid (CSF) | | | | | | | |
| --- | --- | --- | --- | --- | --- | --- | --- |
| *Patient* | *Age (y)* | *Plasma lactate (mmol/L)^a^* | *Plasma pyruvate (mmol/L)^b^* | *Plasma lactate: pyruvate ratio^c^* | *CSF lactate (mmol/L)^d^* | *CSF pyruvate (mmol/L)^b^* | *CSF lactate: pyruvate ratio^c^* |
| P1 | 10 | 1.23 | 0.148 | 8 ↓ | 5.23 ↑ | 0.540 ↑ | 10 |
|  | 17 | 2.62 ↑ | 0.306 | 9 ↓ | - | - | - |
|  | 22 | 2.41 ↑ | - | - | - | - | - |
|  | 27 | 2.66 ↑ | - | - | - | - | - |
| P2 | 10 | 1.33 | 0.206 ↑ | 6 ↓ | 6.51 ↑ | 0.780 ↑ | 8 ↓ |
|  | 17 | 2.17 | 0.159 ↑ | 14 | - | - | - |
|  | 22 | 3.30 ↑ | - | - | - | - | - |
|  | 27 | 2.17 | - | - | - | - | - |

^a^Upper limit of normal (ULN): 2.2 mmol/L. ^b^ULN: 0.150 mmol/L. ^c^Normal range: 10-20. ^d^ULN: 2 mmol/L. Arrows indicate whether the observed value is above or below the range in normal controls.

| **Supplemental Table 2**. Nerve conduction studies and electromyography of P1 (22 years) | | | | | | | |
| --- | --- | --- | --- | --- | --- | --- | --- |
|  | CMAP, mV | MNCV, m/s | DML, ms | F-w, ms | SNAP, µV | SNCV, m/s | EMG |
| Radial nerve | - | **-** | - | - | 19 | 56 | - |
| Ulnar nerve | 11.1 | 51 | 2.5 | 29.7 | **5**^a^ | 52 | N (FDIO) |
| Median nerve | 8.2 | **42** | 3.2 | 30.0 | 10^a^ | **44** | **-** |
| Common peroneal nerve | 8.9 | **39** | 3.8 | 52.1 | - | - | N (TA) |
| Superficial peroneal nerve | - | **-** | - | **-** | **2** | 45 | - |
| Posterior tibial nerve | 11.2 | **39** | 4.3 | 52.4 | - | - | - |
| Sural nerve | - | - | - | - | 11 / 13 | 46 | - |

^a^Orthodromic. [/] = right / left; CMAP = compound muscle action potential; DML = distal motor latency; EMG = needle electromyography; FDIO = first dorsal interosseous muscle; F-w = minimal F wave latency; MNCV = motor nerve conduction velocity; N = normal; SNAP = sensory nerve action potential; SNCV = sensory nerve conduction velocity; TA = tibialis anterior muscle. Values in bold typeface = below control range.

| **Supplemental Table 3**. Nerve conduction studies and electromyography of P2 (22 years) | | | | | | | |
| --- | --- | --- | --- | --- | --- | --- | --- |
|  | CMAP, mV | MNCV, m/s | DML, ms | F-w, ms | SNAP, µV | SNCV, m/s | EMG |
| Radial nerve | - | - | - | - | - | - | - |
| Ulnar nerve | 10.8 | **47** | 3.1 | 31.0 | 8^a^ | **44** | N (FDIO) |
| Median nerve | 8.7 | **47** | 3.7 | 31.4 | 14^a^ | **41** | **-** |
| Common peroneal nerve | 6.2 | **37** | 4.4 | 55.0 | - | - | N (TA) |
| Superficial peroneal nerve | - | - | - | - | - | - | - |
| Posterior tibial nerve | 9.4 | **34** | 3.9 | **60.4** | - | - | - |
| Sural nerve | - | - | - | - | **6** / **7** | 45 | - |

^a^Orthodromic. [/] = right / left; CMAP = compound muscle action potential; DML = distal motor latency; EMG = needle electromyography; FDIO = first dorsal interosseous muscle; F-w = minimal F wave latency; MNCV = motor nerve conduction velocity; N = normal; SNAP = sensory nerve action potential; SNCV = sensory nerve conduction velocity; TA = tibialis anterior muscle. Values in bold typeface = below control range.

| **Supplemental Table 4**. Exome sequencing and variant filtering of patients P1 and P2 | | |
| --- | --- | --- |
|  | *P1* | *P2* |
| Sequencing platform | HiSeq 2000 | HiSeq 2000 |
| Target enrichment system | Roche NimbleGen SeqCap EZ Human Exome v.3.0 | Roche NimbleGen SeqCap EZ Human Exome v.3.0 |
| Alignment tool | Burrows-Wheeler Aligner | Burrows-Wheeler Aligner |
| Variant calling tool | GATK v.1.4-17 | GATK v.1.4-17 |
| Total no. of reads | 58,442,663 | 111,336,958 |
| Aligned reads | 92% | 96% |
| 20x coverage | 51% | 86% |
| 10x coverage | 82% | 92% |
| 2x coverage | 94% | 95% |
| Exonic/splice-site variants | 22335 | 22731 |
| Synonymous variants excluded | 10970 | 11273 |
| MAF <0.1% in the ExAC dataset | 359 | 480 |
| Variants in MitoCarta genes (Supp. Table 4) | 8 | 8 |
| Genes associated with Leigh/Leigh-like syndrome | 1 | 1 |

MAF = minor allele frequency; ExAC = Exome Aggregation Consortium dataset (http://exac.broadinstitute.org); MitoCarta = Human MitoCarta 2.0 (https://www.broadinstitute.org/files/shared/metabolism/mitocarta/human. mitocarta2.0.html).

| **Supplemental Table 5**. DNA sequence variants in MitoCarta genes in patients P1 and P2 (n=9) | | | | | | | | | | | |
| --- | --- | --- | --- | --- | --- | --- | --- | --- | --- | --- | --- |
|  | *Gene* | *Location* | *Variant* | *Zygosity* | *AF* | *MIM* | *Gene-associated Mendelian disorder* | *Inh* | *ClinVar* | *Comments* |  |
| Variants in genes known to cause Leigh syndrome | *PDHA1* | Xp22.12 | NM_000284.3  c.1100A>T; p.(His367Leu) | Het | - | 312170 | Pyruvate dehydrogenase E1-alpha deficiency | XLD | Likely pathogenic | - |  |
| Monoallelic variants in genes known to cause AR disorders | *IARS2* | 1q41 | rs139437119 | Het | 0.044% | 616007 | Cataracts, growth hormone deficiency, sensory neuropathy, sensorineural hearing loss, and skeletal dysplasia? | AR | - | - |  |
|  | *MTPAP* | 10p11.23 | rs201004319 | Het | 0.012% | 613672 | Autosomal recessive spastic ataxia 4? | AR | - | - |  |
|  | *PRODH* | 22q11.21 | rs139160806 | Het | - | 239500 | Hyperprolinemia type 1 | AR | - | - |  |
|  | *AK2* | 1p35.1 | rs1045885332 | Het | - | 267500 | Reticular dysgenesis | AR | Uncertain significance | Only detected in P2; present at a low read depth in WES from P1 |  |
| Monoallelic variants in genes without Mendelian disease asso-ciation | *NME6* | 3p21.31 | rs79445720 | Het | 0.067% | - | - | - | - | Only detected in P1; present at a low read depth in WES from P2 |  |
|  | *SLC25A42* | 19p13.11 | rs200692918 | Het | 0.036% | - | - | - | - | - |  |
|  | *SLC22A4* | 5q31.1 | NM_003059  c.388a>T; p.(Thr130Ser) | Het | - | - | - | - | - | - |  |
|  | *PITRM1* | 10p15.2 | rs28416720 | Het | - | - | - | - | - | Low read depth in WES |  |

AF = allele frequency in ExAC; AR = autosomal recessive; ClinVar = ClinVar archive (https://www.ncbi.nlm.nih.gov/clinvar/); ExAC = Exome Aggregation Consortium dataset (http://exac.broadinstitute.org); Het = heterozygous; Inh = mode of inheritance; WES = whole-exome sequencing; XLD = X-linked dominant.

| **Supplemental Table 6**. *In silico* analysis of the heterozygous missense variant NM_000284.3 (PDHA1): c.1100A>T (p.His367Leu) | | | |
| --- | --- | --- | --- |
|  | | *Score* | *Interpretation* |
| Amino acid change | Grantham | 99 | Moderately conservative |
| Conservation scores | GERP++ | 4.46 | High conservation |
|  | phyloP | 0.82 | Low conservation |
| Predictive algorithms | SIFT | 0.006 | Damaging |
|  | MutPred2 | 0.751 | Deleterious |
|  | PROVEAN | -4.21 | Deleterious |
|  | PANTHER | 750 | Probably damaging |
|  | Align-GVGD | C65 | Likely to interfere with function |
|  | Mutation Assessor | 3.205 | Medium functional impact |
|  | Mutation Taster | 0.999 | Disease causing |
|  | PolyPhen-2 | 0.233 | Benign |
|  | FatHMM | -0.85 | Tolerated |
|  | CADD | 22.6 | Probably deleterious^a^ |

^a^Predicted to be within the 1% most deleterious substitutions in the human genome. Align-GVGD = Align-GVGD class (http://agvgd.iarc.fr/); CADD = Combined Annotation Dependent Depletion v1.3 Phred score (http://cadd.gs.washington.edu/); FatHMM = Functional Analysis through Hidden Markov Models v2.3 weighted score (http://fathmm.biocompute.org.uk/inherited.html); GERP++ = Genomic Evolutionary Rate Profiling RS score (https://genome.ucsc.edu/); Grantham = Grantham Matrix score (reference [1](#_ENREF_1)); Mutation Assessor = Mutation Assessor r3 Functional Impact combined score (http://mutationassessor.org/r3/); Mutation Taster = Mutation Taster-2 probability value (http://www.mutationtaster.org/); MutPred2 = MutPred2 general score (http://mutpred2.mutdb.org/); PANTHER = PANTHER Position Specific Evolutionary Preservation time (http://www.pantherdb.org/tools/csnpScoreForm.jsp?); phyloP = phyloP base-wise conservation score derived from multiple sequence alignment of 46 vertebrate species (https://genome.ucsc.edu/); PolyPhen-2 = Polymorphism Phenotyping v2 HumVar score (http://genetics.bwh.harvard.edu/pph2/); PROVEAN = Protein Variation Effect Analyzer score (http://provean.jcvi.org/); SIFT = Sorting Intolerant From Tolerant algorithm score (http://sift.jcvi.org/).

| **Supplemental Table 7**. Results of X-chromosome inactivation analyses in patients P1 and P2 (see supplementary methods for details) | | | | | | | | | | | |
| --- | --- | --- | --- | --- | --- | --- | --- | --- | --- | --- | --- |
| **Patient** | **CAG allele** | **Length (bp)** | **Peak height / intensity (RFU)** | | | | **Proportion of the inactive short and long alleles^a^** | | | | **X-chromosome inactivation pattern^b^** |
|  |  |  | **Dig.1** | **Dig.2** | **Dig.3** | **Sham** | **Dig.1** | **Dig.2** | **Dig.3** | **Avg.** |  |
| P1 | Short  Long | 386  417 | 4014  1772 | 4712  2122 | 8184  3929 | 15596  8868 | 0.771  0.229 | 0.758  0.242 | 0.754  0.246 | 0.761  0.239 | 76:24 |
| P2 | Short  Long | 386  417 | 3391  598 | 6495  1229 | 6053  1170 | 5981  3543 | 0.563  0.437 | 0.558  0.442 | 0.542  0.458 | 0.554  0.446 | 55:45 |

^a^See supplementary methods for calculation details. Final values were the average (Avg.) of the three restriction digests per subject. ^b^Ratio of the proportion of inactive short allele to the proportion of inactive long allele. bp = base pairs; Dig.1, Dig.2, and Dig.3 = each of the three restriction digests per subject; RFU = relative fluorescence units; Sham = sham digest.

**Supplemental methods**

**Whole-exome sequencing**

The exome targets of the patients’ genomic DNA were captured with SeqCap EZ Human Exome v.3.0 (Roche NimbleGen). The HiSeq 2000 platform (Illumina) was used to produce 100 bp paired-end sequence reads. Reads were aligned to the human genome assembly 19 (GRCh37) with the Burrows-Wheeler Aligner (BWA) tool.[^2^](#_ENREF_2) The Genome Analysis Toolkit (GATK)[^3^](#_ENREF_3) and ANNOVAR[^4^](#_ENREF_4) were used to call and annotate variants. BAM files were visualized with GenomeBrowse v2.1.2 (Golden Helix Inc.).

**Sanger sequencing**

For whole-exome sequencing validation and segregation analysis, the c.1100A>T (p.His367Leu) mutation located in exon 11 of the *PDHA1* gene (NCBI Reference Sequence NG_016781.1; NM_000284.3) was PCR-amplified and Sanger sequenced using the Big Dye Terminator v3.1 Cycle Sequencing Kit (Applied Biosystems), and then analysed on an 3730XL DNA analyser (Applied Biosystems). The resulting electropherograms were examined with SeqScape v2.5 (Applied Biosystems).

**X-chromosome inactivation analysis**

The pattern of X-chromosome inactivation was assessed as previously described.[^5^](#_ENREF_5) In summary, the first exon of the androgen receptor (AR) gene, located in Xq12, contains an in-frame polymorphic CAG repeat encoding a polyglutamine chain. More than 90% of women are heterozygous for this CAG repeat and, in them, differentiation between the maternally and paternally inherited X chromosome is possible through size analysis of the region. Two CCGG sites located less than 100 bp upstream of the CAG repeat are methylated on the inactive X-chromosome in women. Therefore, a restriction digest of female DNA with the methylation-sensitive enzyme HpaII cleaves the active (unmethylated) X chromosome at these CCGG sites and leaves the inactive (methylated) X chromosomes intact. Subsequent PCR of the region leads to amplification of the CAG repeats within the inactive X chromosomes and the relative proportion of each amplified CAG repeat is used as an indicator of the level of inactivation of each X chromosome.

For this study we used DNA extracted from peripheral blood leukocytes since the quantity/quality of DNA extracted from fibroblasts was not sufficient for analysis. Restriction digests were performed using 1 μg DNA and 10 UE of HpaII (New England Biolabs, Inc.) incubated for 12h at 37ºC. For each DNA sample, three restriction digests and one sham-digest with DNA and buffer but no enzyme were performed. A PCR of the digests and sham-digest was then carried out to amplify the CAG repeat region within the AR gene on the inactive X chromosomes. The resulting PCR products were run on a 3730XL DNA sequencer (Applied Biosystems) and analysed with GeneMapper v3.7 (Applied Biosystems). The repeat size (bp) and the peak height (signal intensity proportional to the amount of PCR product) of each amplified CAG repeat allele were obtained. To account for preferential amplification of the allele with the shorter CAG repeat (short allele) vs. the allele with the longer CAG repeat (long allele), peak height values for the digested DNA were normalised with those from the undigested DNA from the sham digest for each subject. The proportion of the inactive short allele was calculated using the formula (PHD_S_/PHU_S_)/(PHD_S_/PHU_S_)+(PHD_L_/PHU_L_), where PHD and PHU are the peak heights of the digested and undigested short (S) and long (L) alleles, respectively, as described by Bittel et al.[^6^](#_ENREF_6) The proportion of the inactive long allele was calculated using the formula (PHD_L_/PHU_L_)/(PHD_S_/PHU_S_)+(PHD_L_/PHU_L_). Final values were the average of the three restriction digests per subject. The X-chromosome inactivation pattern was defined as the ratio of the proportion of the inactive short allele to the proportion of the inactive long allele.

***In silico* analyses**

DNA and protein sequence variants are described in accordance with the recommendations of the Human Genome Variation Society (http://varnomen.hgvs.org/). Evolutionary conservation of nucleotides was assessed using phyloP (46 vertebrate species) and GERP++ scores,[^7^](#_ENREF_7)^,^[^8^](#_ENREF_8) which were accessed through the UCSC Genome Browser (https://genome.ucsc.edu/) using genomic coordinates from human genome assembly GRCh37. Grantham scores were used to assess the physicochemical nature of the amino acid substitution.[^1^](#_ENREF_1) *In silico* analysis of DNA or protein sequence variants was performed using the following pathogenicity prediction tools: Align-GVGD,[^9^](#_ENREF_9)^,^[^10^](#_ENREF_10) CADD,[^11^](#_ENREF_11) FatHMM,[^12^](#_ENREF_12) Mutation Assessor,[^13^](#_ENREF_13)^,^[^14^](#_ENREF_14) Mutation Taster,[^15^](#_ENREF_15) MutPred2,[^16^](#_ENREF_16) PANTHER,[^17^](#_ENREF_17) PolyPhen-2,[^18^](#_ENREF_18)^,^[^19^](#_ENREF_19) PROVEAN[^20^](#_ENREF_20) and SIFT.[^21^](#_ENREF_21)^,^[^22^](#_ENREF_22)

The CABS-flex 2.0 web server[^23^](#_ENREF_23) was employed for the simulation of large-scale structural fluctuations in both wild-type and p.His367Leu enzymes. To this end the coordinates of the holo form of α_2_β_2_-homotetrameric human pyruvate dehydrogenase determined by X-ray crystallography[^24^](#_ENREF_24) were retrieved from the Protein Data Bank (PDB entry 1NI4) and selenomethionines were replaced by methionines. For consistency with UniProt sequences P08559 and P11177, residues were renumbered by addition of 29 and 30 to α and β subunits, respectively, corresponding to the lengths of the transit peptides for mitochondrial localization. The p.His367Leu variant was built in PyMol^[25](#_ENREF_25)^ by replacing p.His367 with the p.Leu rotamer giving rise to the least number of steric clashes.

**References**

1. Grantham R. Amino acid difference formula to help explain protein evolution. Science 1974;185:862-4.

2. Li H, Durbin R. Fast and accurate short read alignment with Burrows-Wheeler transform. Bioinformatics 2009;25:1754-60.

3. McKenna A, Hanna M, Banks E, et al. The Genome Analysis Toolkit: a MapReduce framework for analyzing next-generation DNA sequencing data. Genome Res 2010;20:1297-303.

4. Wang K, Li M, Hakonarson H. ANNOVAR: functional annotation of genetic variants from high-throughput sequencing data. Nucleic Acids Res 2010;38:e164.

5. Siskind CE, Murphy SM, Ovens R, Polke J, Reilly MM, Shy ME. Phenotype expression in women with CMT1X. J Peripher Nerv Syst 2011;16:102-7.

6. Bittel DC, Theodoro MF, Kibiryeva N, Fischer W, Talebizadeh Z, Butler MG. Comparison of X-chromosome inactivation patterns in multiple tissues from human females. J Med Genet 2008;45:309-13.

7. Pollard KS, Hubisz MJ, Rosenbloom KR, Siepel A. Detection of nonneutral substitution rates on mammalian phylogenies. Genome Res 2010;20:110-21.

8. Cooper GM, Stone EA, Asimenos G, et al. Distribution and intensity of constraint in mammalian genomic sequence. Genome Res 2005;15:901-13.

9. Mathe E, Olivier M, Kato S, Ishioka C, Hainaut P, Tavtigian SV. Computational approaches for predicting the biological effect of p53 missense mutations: a comparison of three sequence analysis based methods. Nucleic Acids Res 2006;34:1317-25.

10. Tavtigian SV, Deffenbaugh AM, Yin L, et al. Comprehensive statistical study of 452 BRCA1 missense substitutions with classification of eight recurrent substitutions as neutral. J Med Genet 2006;43:295-305.

11. Kircher M, Witten DM, Jain P, O'Roak BJ, Cooper GM, Shendure J. A general framework for estimating the relative pathogenicity of human genetic variants. Nat Genet 2014;46:310-5.

12. Shihab HA, Gough J, Cooper DN, et al. Predicting the functional, molecular, and phenotypic consequences of amino acid substitutions using hidden Markov models. Hum Mutat 2013;34:57-65.

13. Reva B, Antipin Y, Sander C. Determinants of protein function revealed by combinatorial entropy optimization. Genome Biol 2007;8:R232.

14. Reva B, Antipin Y, Sander C. Predicting the functional impact of protein mutations: application to cancer genomics. Nucleic Acids Res 2011;39:e118.

15. Schwarz JM, Cooper DN, Schuelke M, Seelow D. MutationTaster2: mutation prediction for the deep-sequencing age. Nat Methods 2014;11:361-2.

16. Pejaver V, Urresti J, Lugo-Martinez J, et al. MutPred2: inferring the molecular and phenotypic impact of amino acid variants. bioRxiv 2017;134981.

17. Mi H, Huang X, Muruganujan A, et al. PANTHER version 11: expanded annotation data from Gene Ontology and Reactome pathways, and data analysis tool enhancements. Nucleic Acids Res 2017;45:D183-D9.

18. Adzhubei IA, Schmidt S, Peshkin L, et al. A method and server for predicting damaging missense mutations. Nat Methods 2010;7:248-9.

19. Choi Y, Sims GE, Murphy S, Miller JR, Chan AP. Predicting the functional effect of amino acid substitutions and indels. PLoS ONE 2012;7:e46688.

20. Choi Y, Chan AP. PROVEAN web server: a tool to predict the functional effect of amino acid substitutions and indels. Bioinformatics 2015;31:2745-7.

21. Ng PC, Henikoff S. SIFT: Predicting amino acid changes that affect protein function. Nucleic Acids Res 2003;31:3812-4.

22. Kumar P, Henikoff S, Ng PC. Predicting the effects of coding non-synonymous variants on protein function using the SIFT algorithm. Nat Protoc 2009;4:1073-81.

23. Kuriata A, Gierut AM, Oleniecki T, et al. CABS-flex 2.0: a web server for fast simulations of flexibility of protein structures. Nucleic Acids Res 2018;46:W338-W43.

24. Ciszak EM, Korotchkina LG, Dominiak PM, Sidhu S, Patel MS. Structural basis for flip-flop action of thiamin pyrophosphate-dependent enzymes revealed by human pyruvate dehydrogenase. J Biol Chem 2003;278:21240-6.

25. DeLano WL. The PyMOL molecular graphics system. 1.8.2.0. ed: Schrödinger, LLC; 2015.
