# Supplementary material for: Differential phenotypic expression of a novel PDHA1 mutation in a female monozygotic twin pair
Source: Hum Genet. 2019 Oct 31;138(11):1313–22. doi: 10.1007/s00439-019-02075-9 (PMC6874639; doi:10.1007/s00439-019-02075-9)
Supplement: Supplementary file 2 — Supplementary Figures Legends (DOCX 25 kb) [file 439_2019_2075_MOESM2_ESM.docx]

**Supplementary Figure 1**. Brain MRI of the patients at age 27-28 years. A-C: T2-weighted images (axial view) showing mild peritrigonal white matter signal changes in both patients (A); patchy signal abnormalities in the right lentiform nucleus and a cyst of the velum interpositum in P1 (B); necrotising cavities in both globi pallidi in P2 (B); and signal changes in both cerebellar hemispheres that are more extensive in P2 than in P1 (C). D: FLAIR images (coronal view) showing cerebellar atrophy of the right hemisphere in P1 and bilaterally in P2. E: T1-weighted images (coronal view) demonstrating cerebellar atrophy in P1 and P2 and a globus pallidus lesion in P2.

**Supplementary Figure 2**. Conservation of histidine (H) at amino acid position 367 (red box) of the α subunit of the E1 component of the PDC encoded by the *PDHA1* gene (NM_000284.3, NP_000275.1). A subset of 35 species were chosen, representing the 100 species available at the USCS genome browser (https://genome.ucsc.edu/).

**Supplementary Figure 3**. Overlay of 10 models of wild-type (left) and p.His367Leu variant (right) of the E1 component of the PDC generated with CABS-flex. Each of these ensembles represents selected snapshots from a molecular dynamics simulation and reflects the flexibility of the input structure. Amino acids relevant for the discussion have been labelled.

**Supplementary Figure 4**. Residue fluctuation profile, as calculated by CABS-flex, for the four subunits of wild-type (solid line) and p.His367Leu variant (dotted line) of the E1 component of the PDC. The position of residue 367 in both E1α proteins is highlighted with a marker.

**Supplementary Figure 5**. Differential residue fluctuation (rmsf) profile (p.His367Leu variant–wild-type), as calculated by CABS-flex, for the E1α (green) and E1α’ (magenta) subunits of the E1 component of the PDC.
