# Supplementary figures and images for: Differential phenotypic expression of a novel PDHA1 mutation in a female monozygotic twin pair
Source: Hum Genet. 2019 Oct 31;138(11):1313–22. doi: 10.1007/s00439-019-02075-9 (PMC6874639; doi:10.1007/s00439-019-02075-9)

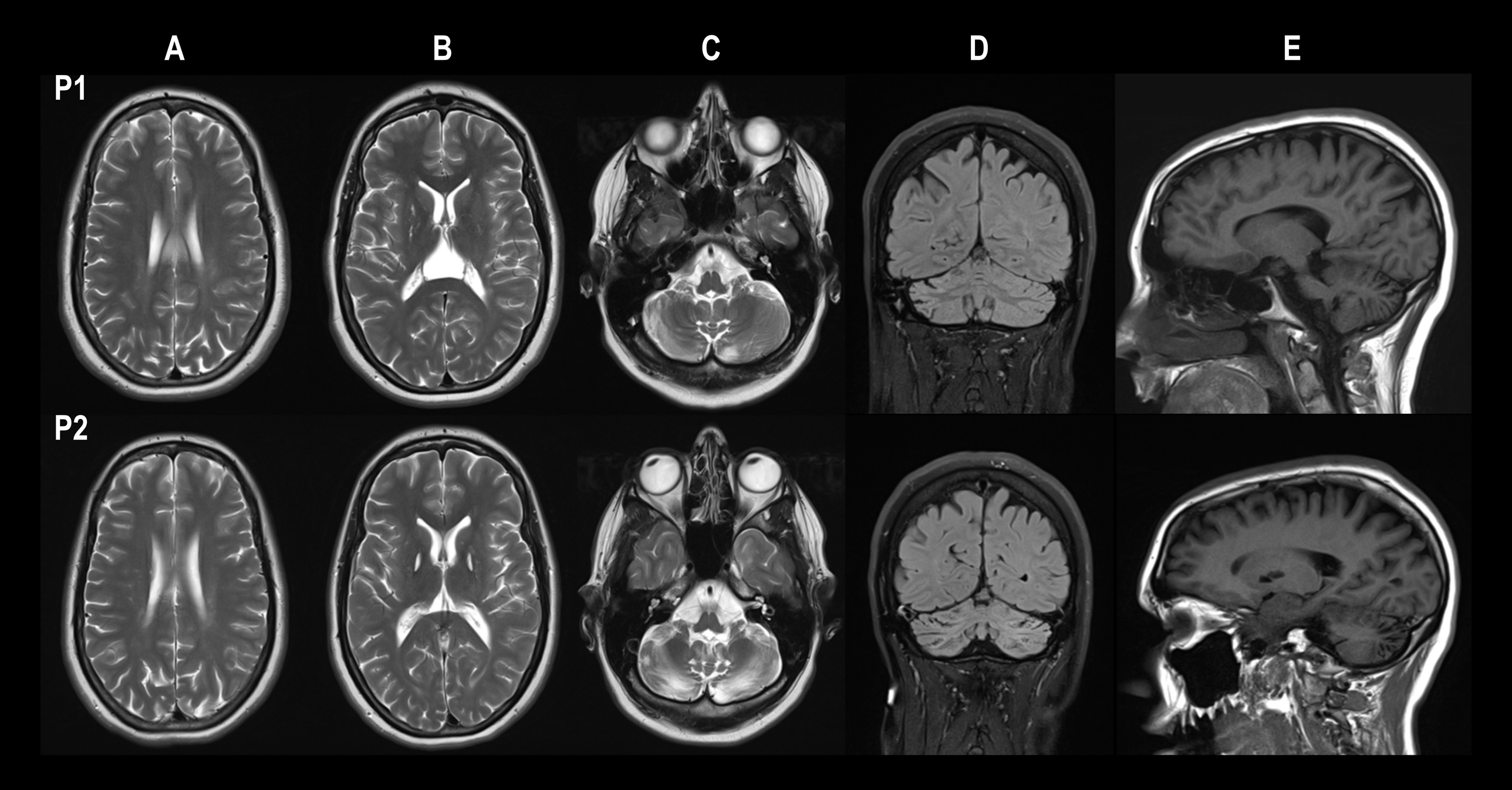

Supplement: Supplementary file 3 — Supplementary Figure 1 (PNG 13078 kb) [file 439_2019_2075_MOESM3_ESM.png]

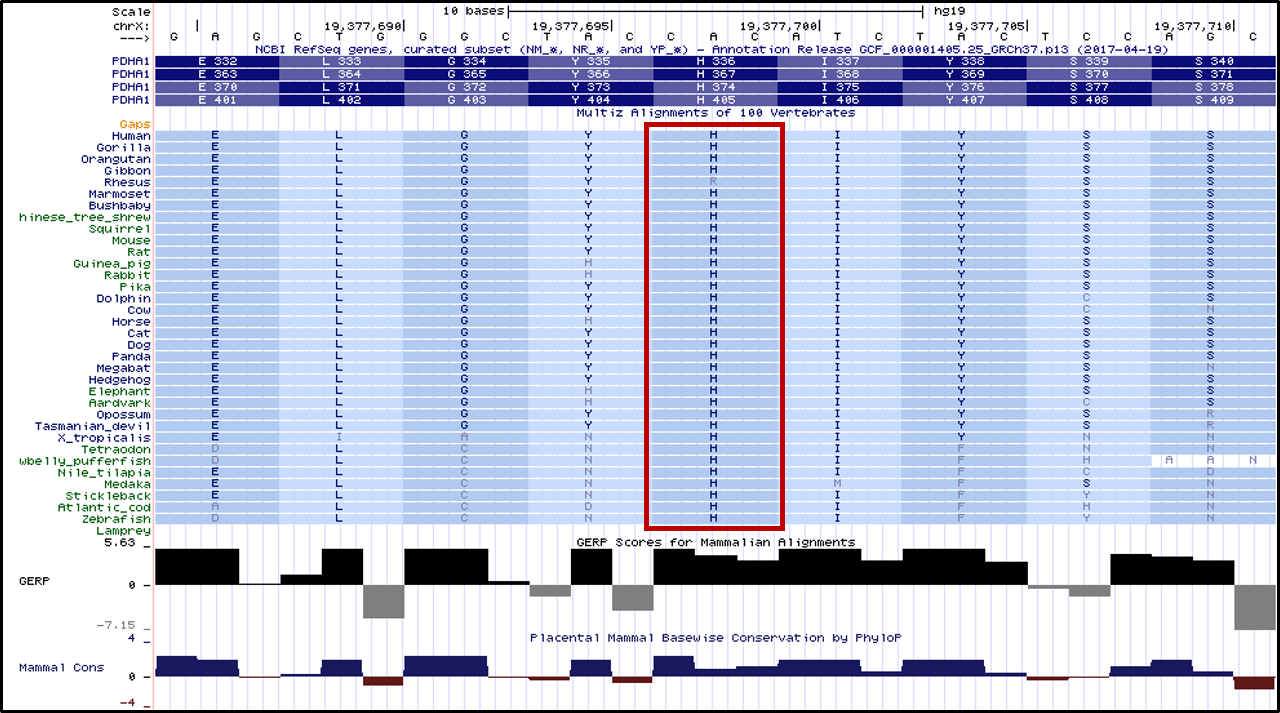

Supplement: Supplementary file 4 — Supplementary Figure 2 (PNG 347 kb) [file 439_2019_2075_MOESM4_ESM.png]

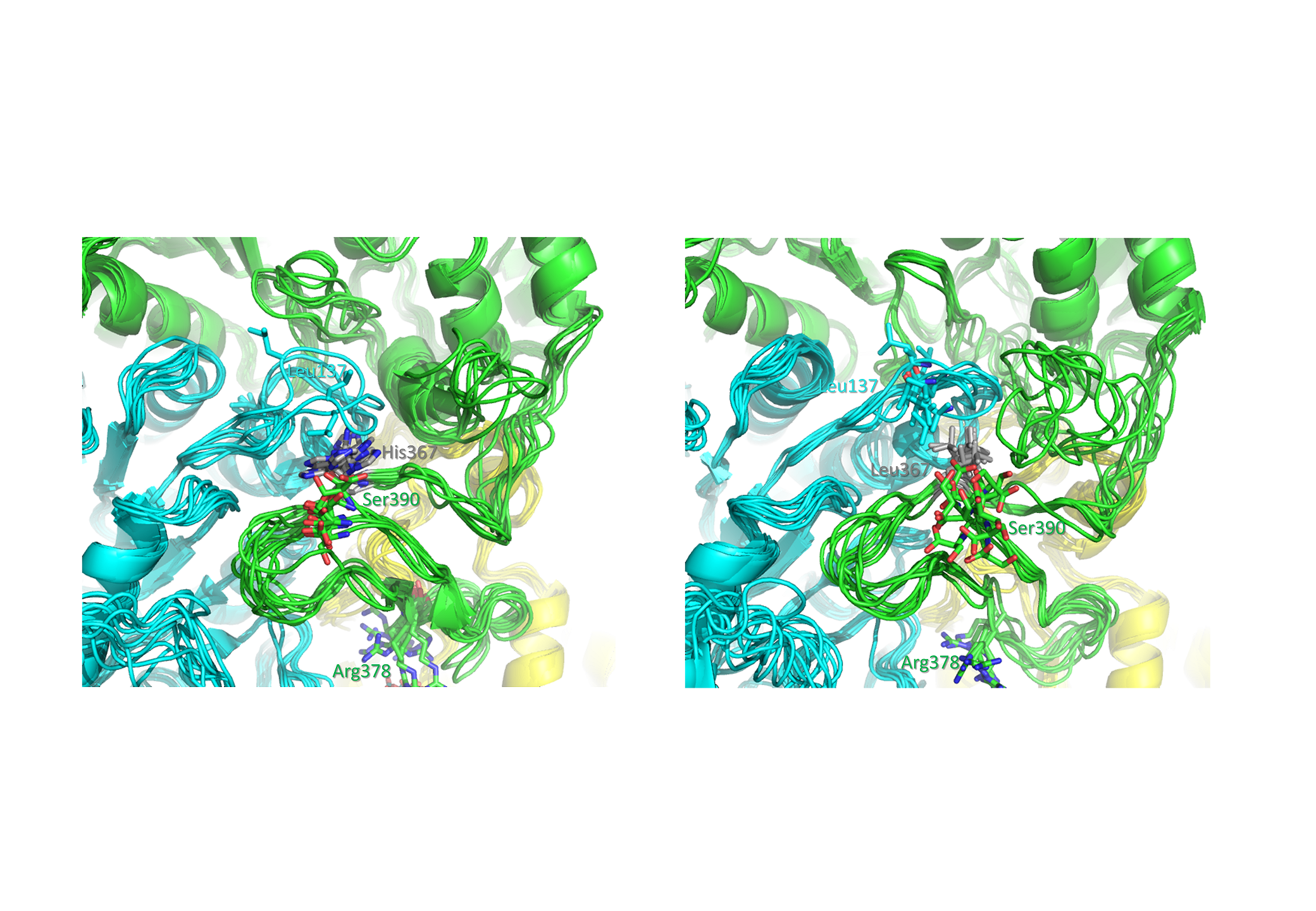

Supplement: Supplementary file 5 — Supplementary Figure 3 (PNG 1984 kb) [file 439_2019_2075_MOESM5_ESM.png]

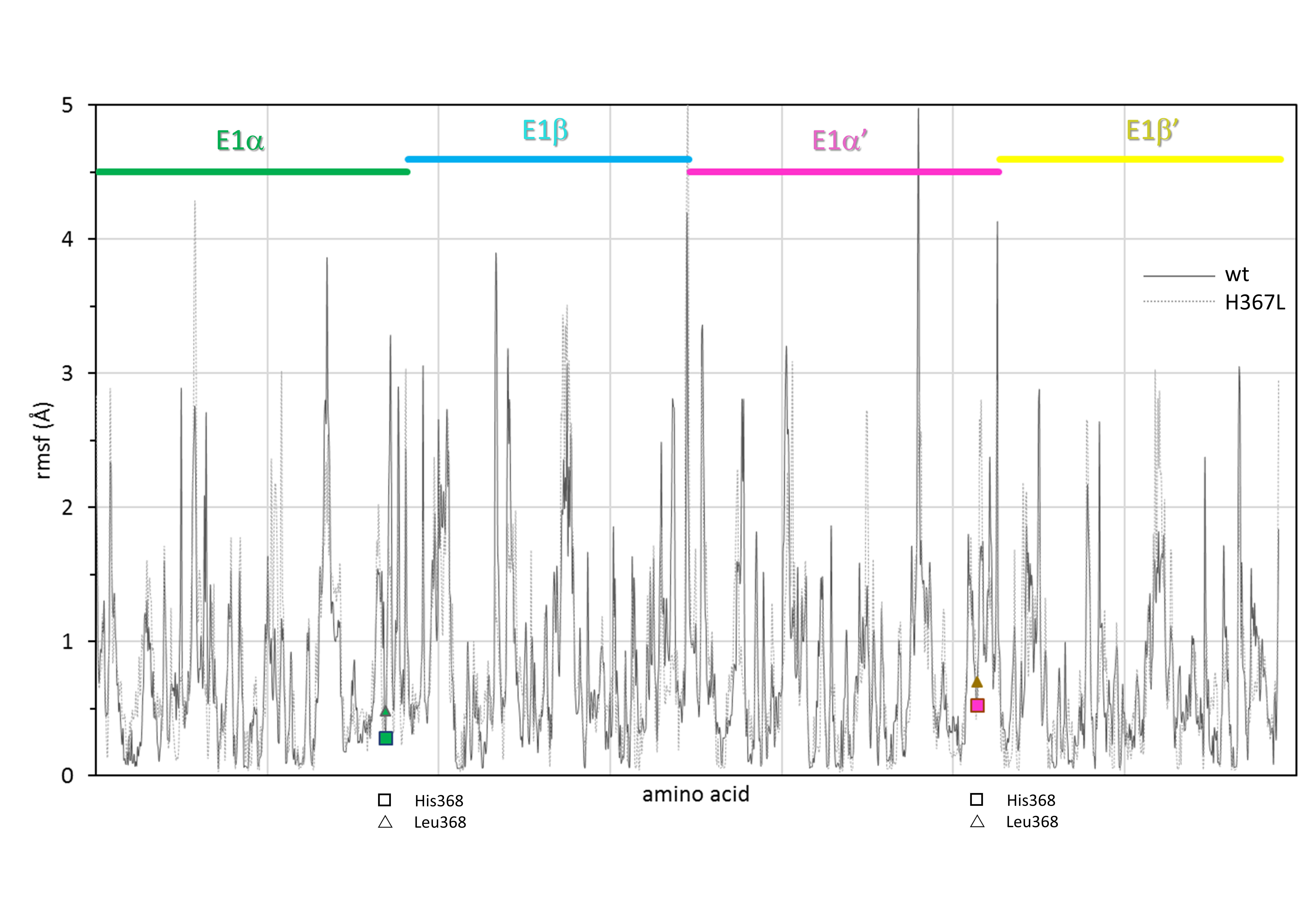

Supplement: Supplementary file 6 — Supplementary Figure 4 (PNG 9571 kb) [file 439_2019_2075_MOESM6_ESM.png]

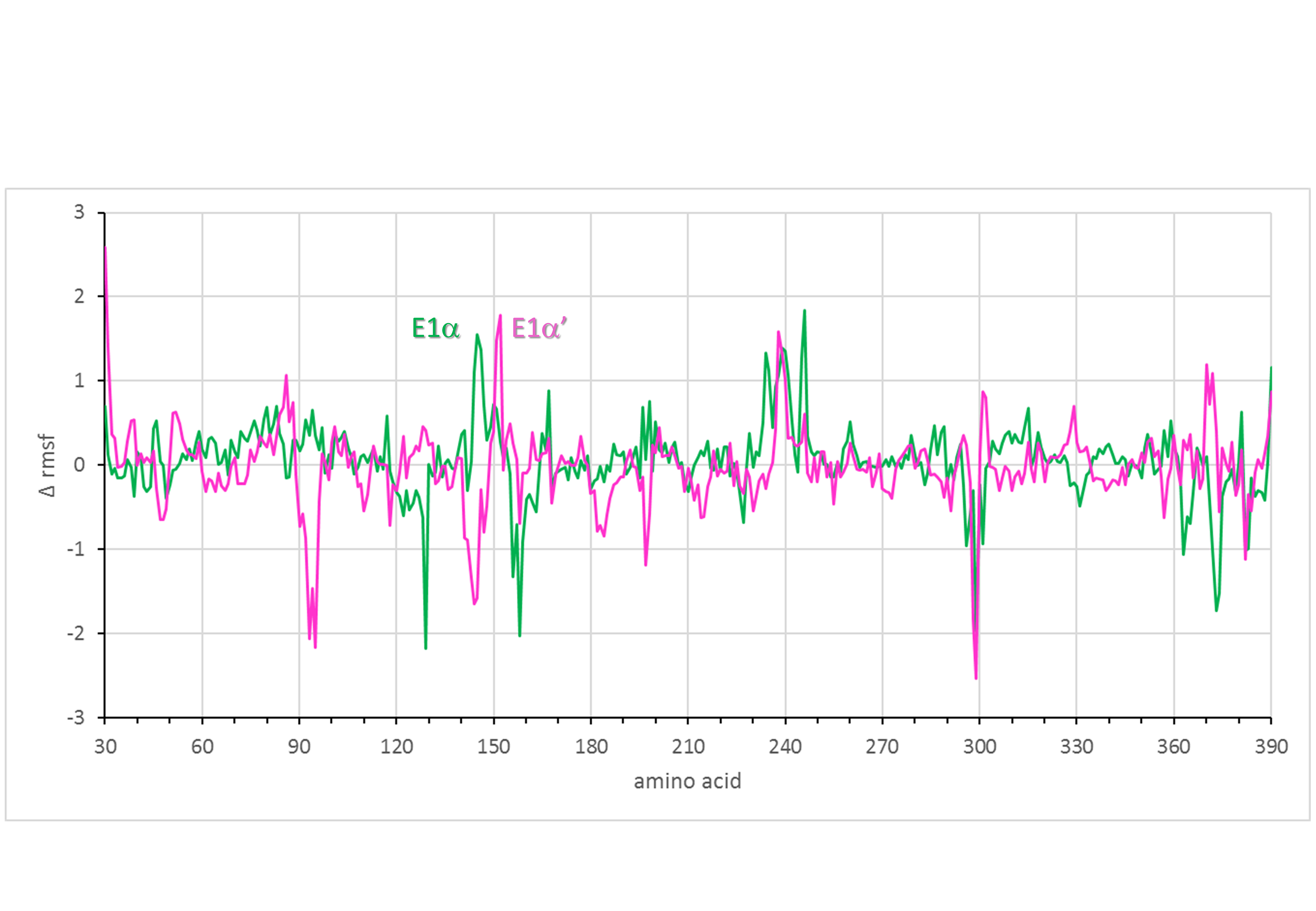

Supplement: Supplementary file 7 — Supplementary Figure 5 (PNG 1256 kb) [file 439_2019_2075_MOESM7_ESM.png]
